# Wakeful Rest Benefits Recall, but Not Recognition, of Incidentally Encoded Memory Stimuli in Younger and Older Adults

**DOI:** 10.3390/brainsci12121609

**Published:** 2022-11-24

**Authors:** Peter R. Millar, David A. Balota

**Affiliations:** 1 Department of Neurology, Washington University in St. Louis, St. Louis, MO 63110, USA; 2 Department of Psychological & Brain Sciences, Washington University in St. Louis, St. Louis, MO 63130, USA

**Keywords:** episodic memory, wakeful rest, consolidation, aging

## Abstract

Older adults exhibit deficits in episodic memory tasks, which have often been attributed to encoding or retrieval deficits, with little attention to consolidation mechanisms. More recently, researchers have attempted to measure consolidation in the context of a behavioral experiment using the wakeful rest paradigm (i.e., a brief, quiet period of minimal stimulation, which facilitates memory performance, compared to a distractor task). Critically, older adults might not produce this effect, given established age differences in other episodic memory processes and mind-wandering. In three experiments, we directly compared younger and older adults in modified versions of the wakeful rest paradigm. Critically, we utilized incidental encoding procedures (all experiments) and abstract shape stimuli (in Experiment 3) to limit the possibility of retrieval practice or maintenance rehearsal as potential confounding mechanisms in producing the wakeful rest effect. Wakeful rest reliably and equally benefited recall of incidentally encoded words in both younger and older adults. In contrast, wakeful rest had no benefit for standard accuracy measures of recognition performance in verbal stimuli, although there was an effect in response latencies for non-verbal stimuli. Overall, these results suggest that the benefits of wakeful rest on episodic retrieval are preserved across age groups, and hence support age-independence in potential consolidation mechanisms as measured by wakeful rest. Further, these benefits do not appear to be dependent on the intentionality of encoding or variations in distractor task types. Finally, the lack of wakeful rest benefits on recognition performance might be driven by theoretical constraints on the effect or methodological limitations of recognition memory testing in the current paradigm.

## 1. Introduction

Proposed models of memory consolidation involve neuronal processes, ranging from the molecular to the systems levels, which stabilize memory traces over time (for review, see [1,2,3,4,5,6], but also see [7,8,9] for alternative perspectives). These consolidation processes are theorized to ultimately transfer the memory trace from hippocampal to neocortical areas, resulting in a stronger long-term trace that is less prone to forgetting. Evidence for consolidation comes from a wide, interdisciplinary range of sources including studies of amnestic individuals (e.g., [10,11]), animal lesion experiments (e.g., [12]), computational modeling of lesioned memory performance [13], experimental manipulations of sleep (for review, see [14]), pharmacological manipulations of specific neurotransmitter agonists and antagonists (e.g., [15,16]), as well as studies of neural replay in animals [17] and humans [18]. Despite this long-standing interest in consolidation within the domains of neuroscience and computational modeling, there have historically been relatively few attempts to manipulate consolidation processes within the span of a behavioral laboratory experiment.

More recently, researchers have developed an intriguing experimental paradigm to examine memory consolidation using “wakeful rest”, a brief period (roughly 10 min) of minimal stimulation while individuals are awake (for review, see [19,20]). In a typical wakeful rest paradigm, participants encode memory stimuli, then often complete an expected *immediate* memory retrieval test for those stimuli. Experimenters then manipulate the intervening period to include either (a) wakeful rest, which takes place alone in a quiet, comfortable room, without access to electronic devices or reading materials, presumably promoting consolidation, or (b) a simple distractor task, such as a spot-the-difference picture game (e.g., [21]), presumably minimizing consolidation. Finally, participants complete a surprise *delayed* retrieval test for all previous memory stimuli. The typical finding is that performance on the delayed retrieval task is better for materials that precede wakeful rest than for those that precede the distractor task. We will henceforth refer to this general behavioral pattern as the “wakeful rest effect”. The wakeful rest effect has been demonstrated using a variety of stimuli, including prose stories [21,22,23], word lists ([24,25], Experiment 1), Icelandic-English word pairs [26,27], pronounceable nonwords ([25], Experiment 2), virtual maps [28,29,30], photographs of everyday objects [31], as well as procedural skill learning [27,32]. In addition, a wide variety of distractor tasks have been employed, including spot-the-difference games, visual search tasks, cued autobiographical recall, and passive listening tasks. Importantly, these distractor tasks often overlap minimally with the memory stimuli in terms of semantic contents, processing demands, and stimulus domains. Thus, one would expect these distractor materials to produce only minimal interference with the memory stimuli. However, wakeful rest effects have been robust, even for these minimally overlapping materials (but see [33]). 

Despite the multiple replications of the wakeful rest effect across a variety of stimulus types and distractor tasks, there have been some recent studies that have failed to observe the expected effect (for review, see [33]). In multiple experiments, Varma and colleagues [34,35] failed to detect a memorial benefit of wakeful rest in comparison to an N-back distractor task, which placed strong cognitive demands on executive resources, but did not overlap with memory stimuli in semantic or autobiographical processing. The authors concluded that, since the N-back task did not demand hippocampal-dependent autobiographical processing, such as other common distractors used in the wakeful rest paradigm, it did not interfere with consolidation processes (but see [36]). Additionally, Martini and colleagues [37] failed to demonstrate a wakeful rest effect in two experiments that used a recall task in the participants’ first language (i.e., German) for prose passages studied in a second proficient language (i.e., English), suggesting that the wakeful rest effect might not be generalizable to a wide range of testing conditions. Humiston and colleagues [38] also demonstrated a failure to replicate the typical wakeful rest effect in a pre-registered replication of an earlier study by Brokaw and colleagues [23], testing free recall of prose passages, but a follow-up meta-analysis suggested that across 10 similar studies, there was a moderate-sized effect (*d* = 0.38) and some of the conflicting results may be driven by sampling error. Most recently, Richter and colleagues [39] reported the absence of wakeful rest benefits on evaluative conditioning. Given these recent failures to extend the wakeful rest effect, it is important to consider whether the effect is robust and generalizable, and moreover, whether the specific boundary conditions may provide insights into the cognitive and neuroscientific mechanisms of the effect.

An important potential moderator of the wakeful rest effect is healthy aging. This factor is of particular interest because the study of age-related differences in episodic memory has typically focused on encoding and retrieval processes, while age differences in consolidation are relatively underexplored (for review, see [40,41,42]). Hence, the wakeful rest paradigm might provide novel insights regarding potential age differences in this understudied stage of memory processing. Early studies have reported the presence of wakeful rest effects independently in samples of healthy older adults [21,25], younger adults [24,26], and even children [43]. More recently, other studies have directly compared age groups in the wakeful rest paradigm. Specifically, Craig and colleagues [29] demonstrated that the wakeful rest benefit for cognitive map accuracy was comparable for both younger and older adults. Critically, however, Martini and colleagues [44] reported an interactive effect of age and condition on word list retention, such that older adults retained more words followed by wakeful rest vs. a distractor condition, whereas younger adults retained a comparable proportion of words across conditions. Most recently, Sacripante and colleagues [45] demonstrated that, in older adults, wakeful rest provided a greater benefit for recall of information related to the gist of a narrative than for peripheral details, however in younger adults, wakeful rest benefited both gist and peripheral information equally. Together, these observations might suggest that rapid consolidation processes for spatial memory, as measured in the wakeful rest paradigm, are relatively preserved in older adults, but consolidation for verbal episodic memory may be age dependent. However, further work is needed to replicate these findings and generalize the interactive age effects to other types of memory stimuli and tests.

The potential age differences in the wakeful rest effect might also offer insight into the role of mind-wandering in memory stabilization. Of course, the task-free conditions during wakeful rest create the opportunity for mind-wandering, and indeed, higher rates of mind-wandering are associated with greater memory enhancement during wakeful rest [23,46] and cued reactivation [47]. Hence, mind-wandering to earlier episodes could serve as retrieval practice and support consolidation type mechanisms. This is particularly important regarding aging since older adults have been repeatedly demonstrated to mind-wander less than younger adults [48,49,50]. Thus, a mind-wandering based account of the wakeful rest effect might predict that older adults should benefit less from wakeful rest than younger adults. However, more careful examination of age differences in the wakeful rest effect is warranted.

A critical, but underexplored, dimension of the wakeful rest paradigm relates to the conditions of memory encoding and expectations of retrieval testing. Specifically, in previous studies, participants have typically encoded stimuli intentionally and were usually aware of at least an immediate memory test before the intervening task, but presumably did not anticipate a surprise delayed test (e.g., [21,24,25]). However, this design may create challenges, as the mere presence of the immediate memory test might increase the likelihood that participants think about the stimuli during the subsequent wakeful rest period. Moreover, the immediate recall test itself might provide an additional opportunity for rehearsal and/or consolidation of memory test items. In line with these alternative interpretations, one recent study demonstrated that long-term (7 day) wakeful rest retention benefits were only observed when retention for the stimuli was also tested on the same day as encoding, but there were no long-term benefits of wakeful rest in the absence of the same-day retrieval test [51]. Moreover, individuals with high working memory capacity have been shown to exhibit a greater wakeful rest benefit for word retention than individuals with low capacity [52]. It is at least possible that high working memory individuals might be more likely to generate a test expectancy than low working memory individuals. This is particularly relevant to the influence of aging, since older adults have lower working memory capacity than younger adults [53]. Thus, under conditions that maximize the expectation of future retrieval testing, wakeful rest benefits might be contaminated by other cognitive processes, including those mediated by working memory capacity, rather than reflecting a “pure” measure of consolidation. Certainly, an unfilled interval after encoding, such as wakeful rest, would afford opportunities to engage in rehearsal, as compared to a condition that minimizes rehearsal via an intervening distractor task that demands the allocation of executive or attentional capacity. Further, because wakeful rest studies often include an immediate retrieval test (before the wakeful rest or distractor period), participants might expect another upcoming memory test, which would encourage some form of rehearsal, or might continue to search for the items that were not recalled on the immediate test [54,55]. This limitation might impact the interpretation of previous age differences in the wakeful rest effect [44], as younger and older adults might engage in these rehearsal or maintenance strategies with differing degrees of success. In order to test whether younger and older adults truly differ in memory consolidation processes, as measured using the wakeful rest paradigm, it is important to test these differences under conditions that minimize the likelihood of rehearsal and other confounding maintenance mechanisms. 

It is important to acknowledge that previous researchers have been aware of rehearsal as a potential confound and have taken steps to control for it. For example, most studies have identified participants who expected the surprise memory test or rehearsed stimuli via a post-task questionnaire, and have shown that the wakeful rest effect is consistent whether or not those participants are included [24,25]. However, since these questionnaires were limited to retrospective self-reports, these responses might be biased by demand characteristics and/or limited in their objective accuracy. Further, Dewar and colleagues ([25], Experiment 2) demonstrated a wakeful rest effect in recognition for nonwords that were presented auditorily under the guise of foreign names, arguing that since these nonwords produced 0% recall accuracy in separate pilot testing, it was impossible for participants to rehearse them. However, since the nonwords were verbally pronounceable, they could be assigned verbal labels, thus potentiating rehearsal, albeit to a low degree of performance see [56,57]. Further, in the experiment by Dewar and colleagues ([25], Experiment 2), the nonwords were presented with explicit encoding instructions to remember the names for a future test. Unlike other similar paradigms, encoding in this experiment was not followed by an immediate memory test, which presumably would serve to limit expectation of the delayed memory test (but see additional concerns above about the influence of an immediate test). In any case, the delayed retrieval test was *not* a surprise to participants, which might have motivated them to engage in rehearsal during the intervening wakeful rest period. Although this study is often considered as evidence that wakeful rest benefits are not dependent upon rehearsal [25], we argue that additional methods are necessary to conservatively rule out the possibility of rehearsal.

In order to further explore whether the wakeful rest effect reflects consolidation processes, and whether these processes are preserved in older adults, we conducted a series of experiments using both rehearsable (words) and non-reheasable (abstract shapes) memory stimuli, under incidental learning conditions in younger and older adults. Critically, incidental encoding represents a key design element to minimize the likelihood of retrieval test expectation and subsequent rehearsal or retrieval practice. To our knowledge, incidental encoding has only been utilized in one previous wakeful rest experiment [31].

**Figure 1:**
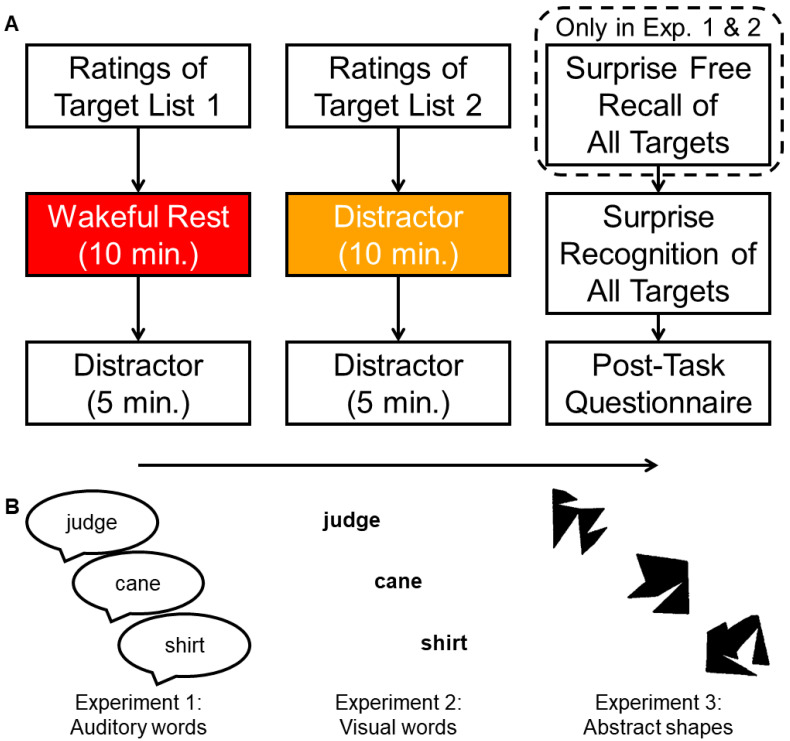
Overall design of Experiments 1–3. (**A**) Diagram of task sequence. (**B**) Example stimuli. Note that Experiment 3 only included a surprise recognition test since abstract shapes were used as stimuli.

Figure 1A depicts the general design of the present experiments. In all experiments, participants encoded memory stimuli via incidental pleasantness ratings. Each encoding block was followed by either a period of wakeful rest or a distractor task, which minimally overlapped with the encoding stimuli in semantic content and stimulus modality. Finally, memory retention was tested with surprise retrieval tasks, followed by a questionnaire to assess whether individuals expected the tests and/or attempted to rehearse the stimuli. Across the three experiments, we explored whether the wakeful rest effect and associated age differences might be further moderated by additional design factors, including the type of the memory stimuli (auditory words [Experiment 1], visual words [Experiment 2], or abstract shapes [Experiment 3]), retrieval tests (free recall [Experiments 1 and 2], or multiple measures of recognition performance, including accuracy [hits-false alarms], hit confidence, and speed of processing [all experiments]), and manipulation of distractor tasks (visual comparison [Experiment 1] or auditory digit monitoring [Experiments 2 and 3]).

## 2. Experiment 1

In Experiment 1, we tested whether the wakeful rest effect was observed for recall and recognition of auditory words that are incidentally encoded, and further, whether the effect differs between older and younger adults. We used a visual spot-the-difference task as a distractor manipulation, in order to preserve minimal overlap in stimulus modality between the memory stimuli and distractor materials, as has been performed in prior wakeful rest experiments using auditory memory stimuli (e.g., [21,25]).

### 2.1. Method

#### 2.1.1. Participants

In all experiments, we used strict retrospective screening criteria to eliminate participants who expected the surprise memory tests, attempted to rehearse memory stimuli, or fell asleep, as determined via self-report on a post-task questionnaire. Critically, participants were informed that compensation would not be determined by their responses to these questions and were encouraged to answer the questionnaire truthfully. These participants were replaced by new participants in the same counterbalance configuration until the final sample was met. In all experiments, we report only analyses on the final sample, excluding the replaced participants. Importantly, for all analyses, the interpretations do not change if we use a more liberal criteria including all participants.

Experiment 1 included a final sample of 24 younger adults and 16 older adults (see Table 1 for demographic characteristics). Younger adults were recruited from the undergraduate participant pool at Washington University in St. Louis (WUSTL) and completed the experiment in exchange for course credit. Older adults were recruited from the Volunteer for Health registry at WUSTL and completed the experiment in exchange for cash payment. Older adults were screened for possible dementia using the Short Blessed Test [58]. All participants scored within the range of normal cognition (0–4 out of 28 [59]), and so no participants were excluded for cognitive impairment.

#### 2.1.2. Design

Experiment 1 included two phases of incidental encoding for auditory words, as well as surprise recall and recognition memory tests (see Figure 1A). Each incidental encoding phase included one unique list of auditory words and was immediately followed by a 10 min intervening period and then a 5 min distractor task. Within participants, we manipulated the activity that filled the post-encoding period (either wakeful rest or a distractor task, counterbalanced across participants). Age group was a between-participant variable (younger or older adults).

#### 2.1.3. Materials

Stimuli included auditory recordings of 60 unrelated nouns, divided into four lists of 15 words. List words were selected from the English Lexicon Project [60] and matched in the distributions of initial letters, total number of letters, number of syllables, log Hyperspace Analogue to Language (HAL) frequency, orthographic neighborhood, phonological neighborhood, and number of phonemes (see Figure 1B). Each recording included the words spoken aloud by a female speaker.

All stimuli were presented using E-Prime 2.0 software (Psychology Software Tools, Pittsburgh, PA, USA). For each participant, two lists of words were selected as stimuli to be presented in the incidental encoding phases, while the other two served as lures in the surprise recognition test. Across participants, each list appeared an equal number of times as a target or as a lure list, an equal number of times in the wakeful rest condition or in the distractor condition, and an equal number of times in the first or second encoding phase.

#### 2.1.4. Procedure

In all experiments, all testing took place in a dimly lit room with minimal furniture and no accessible reading materials. Participants did not have access to phones or any other personal belongings for the duration of the experiment.

After providing informed consent, participants first completed a practice round of a spot the difference task, similar to the one used by Dewar and colleagues [21,25], which was later used as the distractor task. Following the instructions and practice, participants completed two phases of incidental encoding. Participants were instructed through a false cover story that the experiment primarily addressed how words are rated for pleasantness. They were not informed that they would complete any memory tests. In each encoding phase, participants heard 15 words presented over the computer speakers at a comfortable listening volume. Participants were instructed to rate the pleasantness of each word via button press on a Likert scale from 1 (very unpleasant) to 5 (very pleasant) within a 3-s time limit. After each round of pleasantness ratings, the experimenter provided a false cover story that the stimuli in the next round would be selected after the experimenter analyzed the previous pleasantness ratings, thus requiring a break of approximately 10 min. This interval was filled by either wakeful rest or a distractor task, the sequence of which was counterbalanced across participants.

During the 10 min wakeful rest interval, participants were left alone in the testing room. The testing computer was password-locked with a blank monitor, so as not to provide distraction. Participants were instructed to rest quietly and to try not to fall asleep.

During the 10 min distractor interval, participants were also left alone in the testing room and were instructed to complete the spot-the-difference task. During the spot-the-difference task, participants viewed two pictures of the same complex scene, presented side by side. For each picture pair, ten details had been digitally changed between the two images, including the addition or subtraction of small elements, or subtle changes in the color, rotation, or shading of elements. Participants were given 1 min to view each picture pair and identify those differences by mouse click. Participants viewed a total of 10 picture pairs in this manner. 

After each 10 min interval, participants completed a 5 min round of the spot-the-difference task as an additional distractor. This task served to minimize any abrupt carry-over effects of switching from the distractor task to the next encoding list, which may differ between the wakeful rest and distractor conditions. This washout period has been included in some previous wakeful rest experiments (e.g., [25], Experiment 1). Of course, any benefit of wakeful rest on memory consolidation should be present after this 5 min washout period.

After completing both phases of incidental encoding, intervening period, and short distractor tasks, participants completed a surprise recall memory test. Participants were instructed to vocally recall the words they had heard during the pleasantness rating tasks. An experimenter recorded all responses for at least 60 s, and stopped the task when a period of 30 s elapsed without a response. After the recall task, participants completed a recognition memory test for the same words. In the recognition test, participants heard both target words (the two word lists previously presented during the pleasantness rating tasks) and foils (the two word lists held in reserve), which were randomly intermixed. For each of the 60 items, participants were instructed to indicate by button press whether it had been presented in any of the pleasantness rating tasks (i.e., it was “old”) or it was “new”. After each memory judgment, they provided a rating of confidence on a Likert scale from 1 (completely guessing) to 5 (completely confident). Participants had no time limit to provide memory judgments or confidence ratings, but were instructed to do so as quickly and as accurately as possible.

Finally, participants completed a post-task questionnaire. Participants were replaced if their questionnaire responses indicated that they (1) had expected a memory test, (2) attempted to rehearse the words, or (3) fell asleep at any point in the experiment.

#### 2.1.5. Statistical Analysis

Dependent variables of interest included proportion recalled, recognition performance, recognition confidence, and recognition reaction time. Proportion recalled was defined as the number of words recalled from each condition divided by the total number of stimuli (15 per condition). Recognition performance was defined as the proportion of hits for each condition minus the proportion of false alarms. Recognition confidence was defined as the average confidence rating for correct hit trials in each condition. Recognition reaction time (RT) was defined as the average RT for correct hit trials in each condition after excluding outlier trials (more than 3 SD above or below a participant’s mean RT). In order to correct for well-established age differences in overall processing speed, which may confound condition-specific speeding effects [61,62], we standardized RT measurements from all correct recognition hit trials within participants using a z-score transformation to the participant-specific mean and standard deviation of RT [63].

All statistical analyses were performed in R version 4.2.0 [64]. Main effects and interactions of condition and age on memory performance were tested using analysis of variance (ANOVA). Group differences in demographic variables were tested using independent-samples *t* tests for continuous variables or *χ^2^* tests for categorical variables. For all analyses, α was set to 0.05. Effect sizes were reported as generalized Eta squared (*η_G_*^2^).

### 2.2. Results

#### 2.2.1. Questionnaire

Seven younger and 1 older adult reported expecting the memory test. One younger and 1 older adult reported attempting to rehearse the stimuli. Two younger adults reported falling asleep. Each of these participants was replaced by a new participant given the same counterbalancing order of conditions.

#### 2.2.2. Recall

**Figure 2:**
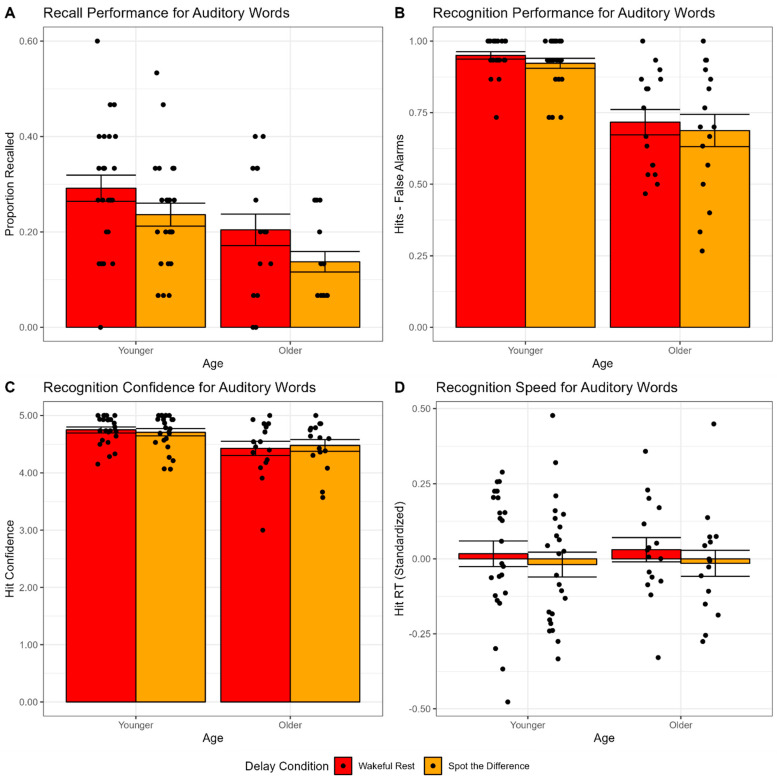
Retrieval task performance measures for auditory word stimuli in younger and older adults in Experiment 1, as a function of delay condition. (**A**) Proportion recalled. (**B**) Recognition performance (hits-false alarms). (**C**) Recognition hit confidence. (**D**) Recognition hit reaction time (standardized). Bar heights reflect mean values. Points reflect individual participants. Error bars are standard error of the mean.

We tested the effects of healthy aging and delay condition on recall performance in a 2 × 2 mixed-model ANOVA, with proportion recalled as the dependent variable, age as a between-participant factor (younger or older adults), and condition as a within-participant factor (wakeful rest or spot the difference). As expected, this analysis revealed a significant main effect of age, *F*(1,38) = 9.07, *p* = 0.005, *η_G_*^2^ = 0.13 and a significant main effect of condition, *F*(1,38) = 6.64, *p* = 0.014, *η_G_*^2^ = 0.06. There was no evidence of an interaction between age and condition, *F*(1,38) = 0.05, *p* = 0.816, *η_G_*^2^ < 0.001. As shown in Figure 2A, older adults recalled fewer words than younger adults did, and both groups recalled more words in the wakeful rest condition than in the spot the difference condition. Similar results were observed if recall performance was corrected for intrusion rates of unstudied words or analyzed as a ratio of relative performance between the two conditions (see Supplementary Materials).

#### 2.2.3. Recognition

We tested the effects of healthy aging and delay condition on recognition performance (defined as hits-false alarms) in a 2 × 2 mixed-model ANOVA with the same factor structure described for recall. This analysis revealed a significant main effect of age, *F*(1,38) = 32.18, *p* < 0.001, *η_G_*^2^ = 0.41, but no main effect of condition, *F*(1,38) = 2.50, *p* = 0.122, *η_G_*^2^ = 0.01, or interaction between age and condition, *F*(1,38) = 0.001, *p* = 0.970, *η_G_*^2^ < 0.001. As shown in Figure 2B, older adults had lower recognition performance than younger adults did, and both groups performed similarly in the wakeful rest condition, as compared to the spot the difference condition.

We tested the effects of healthy aging and delay condition on recognition hit confidence in a 2 × 2 mixed-model ANOVA with the same factor structure described for recall. This analysis revealed a significant main effect of age, *F*(1,38) = 6.49, *p* = 0.015, *η_G_*^2^ = 0.13, but no main effect of condition, *F*(1,38) = 0.006, *p* = 0.941, *η_G_*^2^ < 0.001, or interaction between age and condition, *F*(1,38) = 1.10, *p* = 0.302, *η_G_*^2^ = 0.004. As shown in Figure 2C, older adults had lower confidence ratings than younger adults did, and both groups were similarly confident in the wakeful rest condition, as compared to the spot the difference condition.

We tested the effects of healthy aging and delay condition on recognition speed (standardized within participants) in a 2 × 2 mixed-model ANOVA with the same factor structure described for recall. This analysis revealed no significant main effects of age, *F*(1,38) = 1.61, *p* = 0.213, *η_G_*^2^ = 0.001, or condition, *F*(1,38) = 0.44, *p* = 0.513, *η_G_*^2^ = 0.01, nor an interaction between age and condition, *F*(1,38) = 0.005, *p* = 0.942, *η_G_*^2^ < 0.001. As shown in Figure 2D, after standardizing RTs within participants, there was no difference in RT between the wakeful rest and spot the difference condition for either younger or older adults. Since RTs were standardized within participants, the lack of a main effect of age was not surprising, despite well-established age differences in overall processing speed [61,62].

In order to further test for differential effects of recall vs. recognition, we conducted a 2 (age; between participants; older vs. younger) × 2 (condition; within participants; wakeful rest vs. spot the difference) × 2 (test type: recall vs. recognition accuracy) mixed-model ANOVA. This analysis revealed significant main effects of age (*F*(1,38) = 27.83, *p* < 0.001, *η_G_*^2^ = 0.28), condition (*F*(1,38) = 7.49, *p* = 0.009, *η_G_*^2^ = 0.03), and test type (*F*(1,38) = 930.29, *p* < 0.001, *η_G_*^2^ = 0.85), which were further characterized by a significant age × test type interaction (*F*(1,38) = 8.90, *p* = 0.005, *η_G_*^2^ = 0.05). All other interactions were not significant (*F*s < 1.5, *p*s > 0.20, *η_G_*^2^s < 0.004).

### 2.3. Discussion

The results from Experiment 1 demonstrate the expected wakeful rest effect in recall performance. The recall effect is similar to previous demonstrations (e.g., [21,24,25]), but it is novel in that it extends the effect to incidentally encoded words, which as compared to intentionally encoded stimuli, should minimize participants’ expectations of upcoming retrieval tests and motivation to engage in rehearsal or maintenance. 

Further, we observed an equally large wakeful rest effect on recall for the younger and older adults. This finding is consistent with prior demonstrations of similar wakeful rest effects for auditory word stimuli in independent samples of older adults (e.g., [25]) and younger adults (e.g., [24]). However, the present result conflicts with a recent demonstration of age-dependent benefits of wakeful rest on word list retention [44]. There were a few key methodological differences between the present experiment and the previous report. Specifically, Martini and colleagues [44] used intentional encoding instructions, rather than incidental, and presented word stimuli visually, rather than auditorily. Thus, in Experiment 2, we attempted to replicate this effect using visual word stimuli, to better match the approach of Martini and colleagues [44].

Finally, the wakeful rest effect was not observed in any measure of recognition performance (hits-false alarms, confidence, or speed). It is possible that the failure to observe an effect in recognition performance might be due to a lack of sensitivity in the recognition tests we used and not some theoretical limitation on the wakeful rest effect itself. For instance, recognition performance and confidence exhibited ceiling effects, especially in younger adults (see Figure 2B,C), which may limit sensitivity of these measures to detect an effect of wakeful rest and/or age differences. Importantly, memory retrieval of verbal material has been well established to vary as a function of stimulus modality, with a clear advantage for auditory over visual presentation in both recency effects in short term memory paradigms (for review, see [65]) and learning materials, as supported by a meta-analysis of 43 studies [66], although some individual studies have reported conflicting results [67]. Hence, we aimed to replicate these findings in an independent sample and using a visual stimulus modality, which might elicit performance levels within a lower/more sensitive range of performance.

## 3. Experiment 2

In Experiment 2, we tested whether memory stimuli in a different stimulus modality (i.e., visual, as opposed to auditory, words) would exhibit a wakeful rest benefit, and further, whether the effect differed between older and younger adults. Thus, we aimed to replicate the results of Experiment 1 in an independent sample, while minimizing the limitation of ceiling effects in recognition performance measures.

### 3.1. Method

#### 3.1.1. Participants

Experiment 2 included a final sample of 24 younger adults and 16 older adults, recruited from the same sources described in Experiment 1 (see Table 1). Again, on the Short Blessed Test, all older participants scored within the range of normal cognition, and so no participants were excluded for cognitive impairment.

#### 3.1.2. Design

The design was similar to the one described in Experiment 1 with one major distinction: we tested memory for visually presented words, instead of auditory recordings. Within participants, we manipulated the activity that filled the post-encoding interval (either wakeful rest or a distractor task), with age group as the only between-participant variable. Otherwise, the design was identical to Experiment 1 (see Figure 1A).

#### 3.1.3. Materials

Stimuli included the same lists of nouns from Experiment 1. Words were presented visually in black 24-point Arial font on a white background, instead of auditory recordings (see Figure 1B).

#### 3.1.4. Procedure

The procedure was similar to the one described in Experiment 1. Aspects of the procedure were slightly changed to test the effect in visual word stimuli, as described below.

During each incidental encoding phase, participants viewed words that were presented on the screen for 3 s each and provided pleasantness ratings in a manner similar to Experiment 1.

The post-encoding interval consisted of either wakeful rest or a 10 min interval of a digit monitoring task. In this task, participants listened to audio recordings of digits, ranging from 0 to 9, presented in a pseudorandom order at a rate of one every three seconds. Participants were instructed to press the space bar when they heard an odd digit that was preceded by another odd digit, which occurred on 20% of the trials. After each 10 min interval, participants completed the same digit monitoring task for an additional 5 min.

Apart from the differences described here, the procedure was identical to Experiment 1.

#### 3.1.5. Statistical Analysis

The analytic approach was identical to Experiment 1.

### 3.2. Results

#### 3.2.1. Questionnaire

One younger and 2 older participants reported expecting the memory test. Each of these participants was replaced by a new participant given the same counterbalancing order of conditions.

#### 3.2.2. Recall

**Figure 3:**
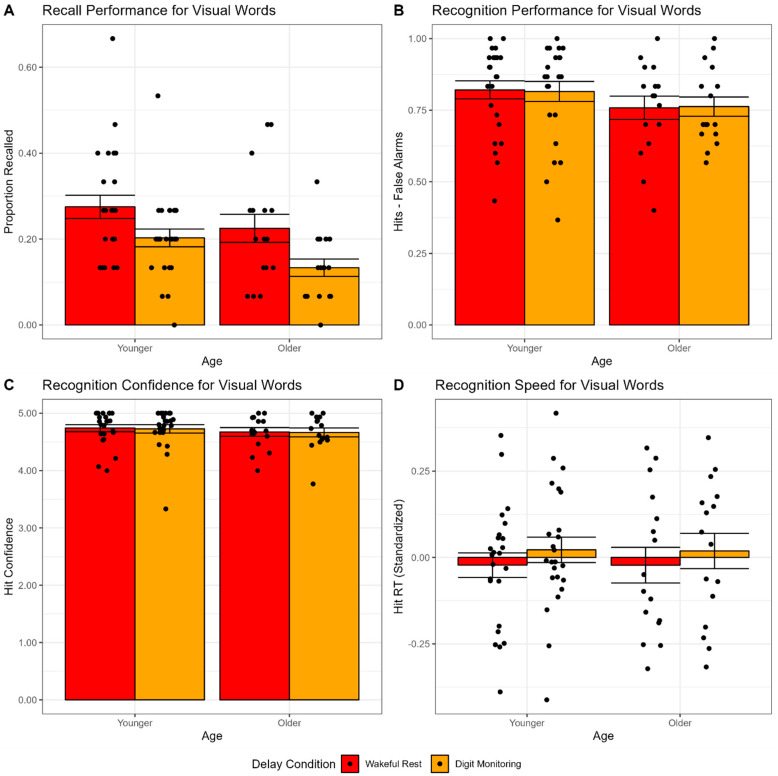
Retrieval task performance measures for visual word stimuli in younger and older adults in Experiment 2, as a function of delay condition. (**A**) Proportion recalled. (**B**) Recognition performance (hits-false alarms). (**C**) Recognition hit confidence. (**D**) Recognition hit reaction time (standardized). Bar heights reflect mean values. Points reflect individual participants. Error bars are standard error of the mean.

The 2 × 2 ANOVA on recall performance (using the same factor structure described in Experiment 1) revealed a significant main effect of condition, *F*(1,38) = 16.89, *p* < 0.001, *η_G_*^2^ = 0.11, but no significant main effect of age, *F*(1,38) = 3.69, *p* = 0.062, *η_G_*^2^ = 0.06, or interaction between age and condition, *F*(1,38) = 0.24, *p* = 0.627, *η_G_*^2^ = 0.002. As shown in Figure 3A, older adults recalled fewer words than younger adults did (although this difference was not statistically significant), and both groups recalled more words in the wakeful rest condition than in the digit monitoring condition. Similar results were observed if recall performance was corrected for intrusion rates of unstudied words (although the main effect of age was significant after correcting for intrusion rates) or analyzed as a ratio of relative performance between the two conditions (see Supplementary Materials). The main effect condition was also significant after controlling for education as a covariate of non-interest, *F*(1,37) = 17.03, *p* < 0.001, *η_G_*^2^ = 0.12, but the main effect of age was not, *F*(1,37) = 2.72, *p* = 0.108, *η_G_*^2^ = 0.05 (see Supplementary Materials for full results).

#### 3.2.3. Recognition

The 2 × 2 ANOVA on recognition performance (hits-false alarms; using the same factor structure described in Experiment 1) revealed no significant main effects of age, *F*(1,38) = 1.46, *p* = 0.234, *η_G_*^2^ = 0.03, or condition, *F*(1,38) = 0.01, *p* = 0.923, *η_G_*^2^ < 0.001, nor an interaction between age and condition, *F*(1,38) = 0.08, *p* = 0.783, *η_G_*^2^ < 0.001. As shown in Figure 3B, older adults had lower recognition performance than younger adults did (although this difference was not statistically significant), and both groups performed similarly in the wakeful rest condition, as compared to the digit monitoring condition.

The 2 × 2 ANOVA on recognition confidence (using the same factor structure described in Experiment 1) revealed no significant main effects of age, *F*(1,38) = 0.44, *p* = 0.510, *η_G_*^2^ = 0.01, or condition, *F*(1,38) = 0.09, *p* = 0.771, *η_G_*^2^ < 0.001, nor an interaction between age and condition, *F*(1,38) = 0.004, *p* = 0.950, *η_G_*^2^ < 0.001. As shown in Figure 3C, older adults recognized words with similar confidence to younger adults, and both groups were similarly confident in the wakeful rest condition, as compared to the digit monitoring condition.

The 2 × 2 ANOVA on recognition RT (standardized within participants; using the same factor structure described in Experiment 1) revealed no significant main effects of age, *F*(1,38) = 0.25, *p* = 0.619, *η_G_*^2^ < 0.001, or condition, *F*(1,38) = 0.52, *p* = 0.475, *η_G_*^2^ = 0.01, nor an interaction between age and condition, *F*(1,38) = 0.001, *p* = 0.980, *η_G_*^2^ < 0.001. As shown in Figure 3D, after standardizing RTs within participants, there was no difference in RT between the wakeful rest and digit monitoring conditions for either younger or older adults. Again, since RTs were standardized within participants, the lack of a main effect of age was not surprising, despite well-established age differences in overall processing speed [61,62].

Since age groups differed in years of education, we also tested these effects using ANCOVAs, which included education as a covariate of non-interest. The overall interpretations were largely consistent, including no significant main effects of condition or age × condition interactions for hits-false alarms (*F*s = 0.01, *p*s > 0.9, *η_G_*^2^s < 0.001), confidence (*F*s < 0.03, *p*s > 0.8, *η_G_*^2^s < 0.001), or ZRT (condition: *F*(1,37) = 1.24, *p* = 0.272, *η_G_*^2^ = 0.03; interaction: *F*(1,37) = 0.11, *p* = 0.744, *η_G_*^2^ = 0.003). However, the main effect of age on confidence was not significant after controlling for education, *F*(1,37) = 0.24, *p* = 0.624, *η_G_*^2^ = 0.006 (see Supplementary Material for full results).

In order to further test for differential effects of recall vs. recognition, we conducted a 2 (age; between participants; older vs. younger) × 2 (condition; within participants; wakeful rest vs. digit monitoring) × 2 (test type: recall vs. recognition accuracy) mixed-model ANOVA. This analysis revealed significant main effects of age (*F*(1,38) = 4.68, *p* = 0.037, *η_G_*^2^ = 0.06), condition (*F*(1,38) = 8.12, *p* = 0.007, *η_G_*^2^ = 0.02), and test type (*F*(1,38) = 632.73, *p* < 0.001, *η_G_*^2^ = 0.83), which were further characterized by a significant condition × test type interaction (*F*(1,38) = 11.66, *p* = 0.002, *η_G_*^2^ = 0.02). All other interactions were not significant (*F*s < 0.4, *p*s > 0.50, *η_G_*^2^s < 0.003).

### 3.3. Discussion

The results from Experiment 2 demonstrate that recall for incidentally encoded visual word stimuli, like auditory words in Experiment 1, benefits from a post-encoding period of wakeful rest. Once again, the recall effect was consistent for both younger and older adults. This result contrasts with the recent report of age-dependent effects of wakeful rest on retention for intentionally encoded visual word lists [44]. However, as we have presently replicated the effect using visual memory stimuli, the primary methodological distinction between the present experiments and Martini and colleagues [44] is the encoding instructions (intentional vs. incidental). We will return to this issue in the General Discussion.

The present failure to observe a wakeful rest effect in recognition performance is inconsistent with some previous studies [25,31] and poses a problem for theoretical interpretation of the present results. Although the distribution of recognition performance was less restricted by ceiling effects (Figure 3B) than in Experiment 1 (Figure 2B), the confidence ratings in Experiment 2 still demonstrated an apparent ceiling effect (Figure 3C), which may limit sensitivity of this measure. However, no effect of wakeful rest was observed on processing speed, which is of course not limited by ceiling effects in the present results and has been shown to be sensitive to experimental manipulation and age differences in a similar cuing paradigm [47]. Thus, in order to further explore this issue, we aimed to test for wakeful rest effects and age differences in recognition memory performance using a design intended to increase difficulty (thus examining a different range of the recognition performance and confidence distributions, less restricted by ceiling effects), while at the same time minimizing the chances of rehearsal.

## 4. Experiment 3

In Experiment 3, we tested whether abstract shapes, as opposed to verbal word list stimuli, would exhibit a wakeful rest benefit. We anticipated that recognition performance for these stimuli would be poorer than for verbal stimuli, and thus, we would eliminate ceiling effect constraints from recognition performance and confidence measures. Further, these materials lack a reliable verbal label, and are less readily rehearsable than verbal stimuli. Thus, we tested whether measures of recognition memory performance are sensitive to the wakeful rest effect (and potential interactions with age) under conditions that minimize the possibility of rehearsal. 

### 4.1. Method

#### 4.1.1. Participants

Experiment 3 included a final sample of 32 younger adults and 32 older adults, recruited from the same sources described in Experiment 1 (see Table 1). Again, on the Short Blessed Test, all older participants scored within the range of normal cognition, and so no participants were excluded for cognitive impairment.

#### 4.1.2. Design

The design was similar to Experiment 1 with one major distinction: we tested memory for abstract shapes instead of word stimuli. Within participants, we manipulated the activity that filled the post-encoding interval (either wakeful rest or a distractor task). Age group was a between-participant variable (younger or older adults). Otherwise, the design was identical to Experiment 1 (see Figure 1A).

#### 4.1.3. Materials

We selected 60 abstract shapes that were randomly generated and normed by Vanderplas and Garvin [68]. These shapes were divided into four lists of 15 shapes. Lists were matched in the distributions of total number of points and association value (i.e., the degree to which the shape reminded participants of something meaningful, according to Vanderplas & Garvin [68]), such that each shape had a similar lure in each of the other lists. Shapes were presented filled in with black on a white background (see Figure 1B).

#### 4.1.4. Procedure

The procedure was similar to Experiment 2. Aspects of the procedure were slightly changed to test the effect using abstract shape stimuli instead of words, as described below.

During incidental encoding, participants viewed a series of abstract shapes for 3 s each and were instructed to provide pleasantness ratings, similar to Experiment 2.

The post-encoding interval consisted of either wakeful rest or a 10 min interval of the digit monitoring task from Experiment 2. After each 10 min interval, participants completed the same digit monitoring task for an additional 5 min.

#### 4.1.5. Statistical Analysis

The analytic approach was similar to Experiment 1. However, as there was no recall test for abstract shapes, we tested measures of recognition memory only (hits-false alarms, confidence, and standardized reaction time).

### 4.2. Results

#### 4.2.1. Questionnaire

Eight younger and 13 older participants reported some expectancy for a memory test. Four older participants reported attempting to rehearse the stimuli. Three younger and 2 older participants reported falling asleep. Each of these participants was replaced by a new participant given the same counterbalancing order of conditions. 

#### 4.2.2. Recognition

**Figure 4:**
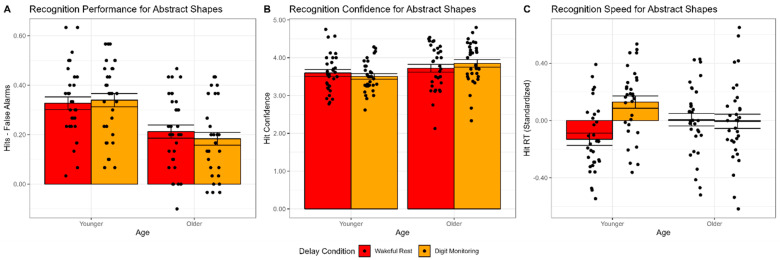
Retrieval task performance measures for abstract shape stimuli in younger and older adults in Experiment 3, as a function of delay condition. (**A**) Recognition performance (hits-false alarms). (**B**) Recognition hit confidence. (**C**) Recognition hit reaction time (standardized). Bar heights reflect mean values. Points reflect individual participants. Error bars are standard error of the mean.

The 2 × 2 ANOVA on recognition performance (hits-false alarms; using the same factor structure described in Experiment 1) revealed a significant main effect of age, *F*(1,62) = 19.00, *p* < 0.001, *η_G_*^2^ = 0.18, but no significant main effect of condition, *F*(1,62) = 0.17, *p* = 0.680, *η_G_*^2^ < 0.001, nor an interaction between age and condition, *F*(1,62) = 1.07, *p* = 0.304, *η_G_*^2^ = 0.005. As shown in Figure 4A, older adults had lower recognition performance than younger adults, and both groups performed similarly in the wakeful rest condition, as compared to the digit monitoring condition.

The 2 × 2 ANOVA on recognition confidence (using the same factor structure described in Experiment 1) revealed a significant main effect of age, *F*(1,61) = 4.29, *p* = 0.043, *η_G_*^2^ = 0.05, but no significant main effect of condition, *F*(1,61) = 0.09, *p* = 0.769, *η_G_*^2^ < 0.001, nor an interaction between age and condition, *F*(1,61) = 2.71, *p* = 0.105, *η_G_*^2^ = 0.01. As shown in Figure 4B, older adults, surprisingly, recognized words with greater confidence than younger adults. Older adults had slightly greater confidence in the digit monitoring condition, as compared to the wakeful rest condition, while younger adults trended in the opposite direction, but this interaction was not statistically significant.

Finally, the 2 × 2 ANOVA on recognition RT (standardized within participants; using the same factor structure described in Experiment 1) revealed no significant main effect of age, *F*(1,61) = 0.02, *p* = 0.902, *η_G_*^2^ < 0.001, or condition, *F*(1,61) = 3.80, *p* = 0.056, *η_G_*^2^ = 0.057, but a significant interaction between age and condition, *F*(1,61) = 4.67, *p* = 0.035, *η_G_*^2^ = 0.069. As shown in Figure 4C, after standardizing RTs within participants, younger adults were significantly faster to recognize words in the wakeful rest condition, as opposed to the digit monitoring condition, *F*(1,30) = 9.29, *p* = 0.005, *η_G_*^2^ = 0.234, but this difference was absent in older adults, *F*(1,31) = 0.01, *p* = 0.905, *η_G_*^2^ < 0.001. Since RTs were standardized within participants, the lack of a main effect of age was not surprising, despite well-established age differences in overall processing speed [61,62].

Since age groups differed in years of education, we also tested these effects using ANCOVAs, which included education as a covariate of non-interest. The overall interpretations were largely consistent, including a significant main effect of condition and age × condition interaction for ZRT (condition: *F*(1,57) = 4.56, *p* = 0.037, *η_G_*^2^ = 0.07; interaction: *F*(1,57) = 4.74, *p* = 0.034, *η_G_*^2^ = 0.08), but not for hits-false alarms (*F*s < 1.00, *p*s > 0.3, *η_G_*^2^s < 0.005). However, the effects on confidence were somewhat different after controlling for education, including a non-significant main effects of age, *F*(1,57) = 2.42, *p* = 0.125, *η_G_*^2^ = 0.03, and condition, *F*(1,57) = 0.17, *p* = 0.680, *η_G_*^2^ = 0.001, but an age × condition interaction of *F*(1,57) = 4.00, *p* = 0.050, *η_G_*^2^ = 0.01 (see Supplementary Material for full results).

### 4.3. Discussion

The results from Experiment 3 demonstrate that recognition memory performance and confidence for abstract shapes does not benefit from a post-encoding period of wakeful rest. This effect is inconsistent with a previous demonstration of a wakeful rest effect for recognition of intentionally encoded nonword stimuli, which the authors argued were non-rehearsable ([25], Experiment 2). However, the present results are also consistent with the absence of wakeful rest effects in measures of recognition memory performance that we observed in Experiments 1 and 2. Importantly, younger and older adults did not demonstrate contrasting effects of wakeful rest for recognition performance or confidence.

Surprisingly, the interactive effect between age and condition on hit reaction time might suggest a possible age-dependent memory benefit of wakeful rest. However, similar speeding effects were not observed for word stimuli in Experiments 1 and 2, nor are we aware of other prior studies that have reported similar speeding effects in recognition hit reaction time, although response latencies have typically not been reported in previous studies. Thus, this effect should be interpreted with caution. However, it is worth noting that similar speeding effects were recently demonstrated by Nicosia and Balota [47] using a cuing paradigm for recently encoded materials. Moreover, the speeding effects showed a similar age interaction in that the effects of cuing during the retention interval led to greater speeding on cued recall for younger adults than for older adults [47].

## 5. General Discussion

Across three experiments investigating the wakeful rest effect, we observed three consistent patterns. First, with the exception of reaction time speeding for abstract shape recognition in Experiment 3, wakeful rest benefits on memory performance did not interact with age group for any measure of memory performance (Experiments 1–3). Second, wakeful rest benefits were consistently observed for recall of incidentally encoded verbal stimuli (Experiments 1 and 2). Third, wakeful rest benefits were consistently not observed for recognition of verbal or non-verbal stimuli (Experiments 1–3).

In order to further increase the power of the statistical tests, we conducted a supplementary ANOVA combining recognition performance (hits-false alarms) from all three experiments, which included experiment as an additional between-participants factor. This analysis revealed significant main effects of experiment, *F*(1,138) = 294.36, *p* < 0.001, *η_G_*^2^ = 0.771, and age group, *F*(1,138) = 40.1, *p* < 0.001, *η_G_*^2^ = 0.198, but no evidence of a main effect of delay condition in the merged dataset, *F*(1,138) = 1.13, *p* = 0.289, *η_G_*^2^ = 0.002, nor an interaction between experiment and condition on recognition performance across the three experiments, *F*(1,138) = 0.45, *p* = 0.637, *η_G_*^2^ = 0.001. Thus, although the small samples within each experiment limit the interpretation of null effects in the recognition data, these null effects are consistently observed in the combined dataset across three experiments.

Finally, these overall patterns were also supported by another supplementary ANOVA, which combined recall (after correcting for intrusion rates, see Supplementary Material) and recognition performance (hits-false alarms) from Experiments 1 and 2, and included test type (recall vs. recognition) as a within-participants factor and experiment as a between-participants factor. Critically, this analysis revealed a significant interaction between wakeful rest condition and test type, *F*(1,76) = 10.00, *p* = 0.002, *η_G_*^2^ = 0.010, but no three-way interaction between age, condition, and test type, *F*(1,76) = 0.30, *p* = 0.585, *η_G_*^2^ < 0.001. Follow-up comparisons revealed that the main effect of wakeful rest condition was significant across both experiments for recall tests, *F*(1,76) = 21.29, *p* < 0.001, *η_G_*^2^ = 0.071, but not for recognition tests, *F*(1,76) = 1.46, *p* = 0.230, *η_G_*^2^ = 0.003.

We now discuss each of these results in turn, with a particular focus on how our observations fit with the previous literature and how they inform age differences in consolidation processes, as well as theoretical interpretations of the wakeful rest effect.

### 5.1. Consistent Wakeful Rest Effects for Younger and Older Adults

In Experiments 1 and 2, we found that the benefits of wakeful rest for verbal recall were similar for both younger and older adults. This observation is consistent with previous demonstrations of wakeful rest benefits in independent samples of older (e.g., [25]) and younger adults (e.g., [24]). It is also consistent with at least one direct comparison of the wakeful rest effect between younger and older adults for spatial learning. Specifically, Craig and colleagues [29] found that younger and older adults exhibited comparable benefits of wakeful rest in map learning accuracy, as assessed using a virtual landmark pointing task. Together with the present results, these findings suggest that the processes involved in the wakeful rest memory effect might be relatively preserved in advancing age for both spatial and episodic memory systems. If this effect indeed reflects the consolidation stage of memory processing, this preservation would stand in contrast to many well-documented age-related deficits in other episodic memory processes (for review, see [40,41,42]).

The present findings are inconsistent, however, with a recent demonstration of age-dependent wakeful rest effects [44]. Specifically, Martini and colleagues [44] observed the presence of a wakeful rest effect for word list retention in older adults, but not in younger adults. In contrast to the present methods, participants in the prior study encoded verbal stimuli intentionally. Given recent demonstrations that the benefits of wakeful rest on retention are moderated by the presence of intermediate retrieval tests [51], as well as working memory capacity [52], it is possible that the age-dependent results reported by Martini and colleagues [44] are driven by factors related to test expectancy and/or working memory maintenance, which would be more likely to occur in the context of intentional, as opposed to incidental encoding. Indeed, older adults might be more conscientious and/or motivated to perform well on the memory task, as compared to younger adults [69,70], but of course, other studies have found that older adults are less likely to initiate effective memory strategies [71,72,73,74]. In the present study, we did not notice a consistent pattern of age differences in self-reported test expectancy in Experiment 1 (Younger: 20.6% vs. Older: 5.6%; two-proportion *z* = 1.43, *p* = 0.153), Experiment 2 (Younger 2.9% vs. Older: 11.1%; two-proportion *z* = −1.23, *p* = 0.219), or Experiment 3 (Younger: 18.6% vs. Older 25.5%; two-proportion *z* = −0.80, *p* = 0.424). Alternatively, the present experiments might also be limited by relatively small sample sizes, and thus might have lower power to detect an interactive effect than Martini and colleagues [44]. Specifically, Experiments 1 and 2 achieved 80.7% power to detect an interactive effect of *η*^2^ ≥ 0.05, but only 23.6% power to detect smaller effects (*η*^2^ ≥ 0.01) [75]. Hence, as noted above, we performed a supplementary analysis combining data from Experiments 1 and 2 to form a combined sample of 32 older adults and 48 younger adults, which is comparable to the final sample of 32 older and 40 younger adults in [44]. A supplementary ANOVA, which included experiment as an additional between-participants factor, revealed significant main effects of distractor condition, *F*(1,76) = 21.29, *p* < 0.001, *η_G_*^2^ = 0.085, and age group, *F*(1,76) = 12.15, *p* < 0.001, *η_G_*^2^ = 0.096, but no evidence of an interaction between age group and condition on recall performance across the two experiments, *F*(1,76) = 0.24, *p* = 0.623, *η_G_*^2^ = 0.001, nor a main effect difference between the two experiments, *F*(1,76) = 0.30, *p* = 0.88, *η_G_*^2^ = 0.003. Thus, a difference in experiment-specific statistical power does not explain the inconsistent results between the two studies.

In Experiment 3, we found that younger adults were faster to correctly identify abstract shapes that preceded wakeful rest, in comparison to a distractor task, but this speeding effect was not present in older adults. It is possible that the speeding effect observed in younger adults in the wakeful rest condition may represent a strengthening of memory traces for the abstract shape stimuli. However, to our knowledge, no prior study has demonstrated a similar benefit of wakeful rest on speeding for episodic retrieval, nor were any similar effects observed in Experiments 1 or 2. Thus, the speeding effect should be interpreted carefully. However, we have recently shown that similar age-dependent speeding effect are consistently observed in the same expected direction for cued recall performance using a targeted memory cuing paradigm during a post-encoding retention interval [47]. Inconsistencies in the presence of the speeding effect may be related to levels of overall performance, characteristics of the stimuli, and/or aspects of the testing procedure. Specifically, it is possible that speeding effects might not be observed in Experiments 1 or 2 due to the high (near-ceiling) levels of performance, but are observed when recognition performance is lower (as in Experiment 3). Alternatively, speeding effects may possibly be dependent upon the stimulus modality or degree of novelty vs. familiarity. The abstract shapes in Experiment 3 are notably much less familiar than the word stimuli in Experiments 1 and 2. Finally, the recognition speeding effect may possibly be influenced by the presence (as in Experiments 1 and 2) or absence (Experiment 3) of a recall test preceding recognition. Thus, these findings should motivate future studies to more carefully examine additional metrics of retrieval performance beyond recall or recognition accuracy (including reaction time, which has not typically been reported) to measure the memorial enhancements of wakeful rest and/or targeted reactivation, as well as the factors that modulate these effects.

### 5.2. Benefit of Wakeful Rest on Recall for Incidentally Encoded Verbal Stimuli

The vast majority of previous wakeful rest experiments have used a paradigm in which verbal materials are intentionally encoded for an immediate recall test, but then memory for the same materials is also tested after a wakeful rest and/or distractor task in a surprise delayed recall test (e.g., [21,24,25]). Prior authors have argued that, because there is an immediate recall test, participants should no longer expect a memory test, and so should be less likely to rehearse or think about the items. However, one might argue that the presence of an immediate memory test might produce the opposite effect. Specifically, under these circumstances, the presence of the immediate recall test might signal the importance of memory for the stimuli, encouraging participants to engage in rehearsal of successfully recalled stimuli during the subsequent wakeful rest interval. Moreover, participants might also be motivated to continue searching their memory for stimuli that were *not* successfully recalled in the immediate test. These behaviors could contribute to the wakeful rest memory effect through mechanisms that are mediated by working memory capacity (which is reduced in older adults [76]) and/or controlled retrieval, rather than automatic, spontaneous consolidation processes. Importantly, however, in Experiments 1 and 2 we found beneficial effects of wakeful rest on recall for words that were incidentally encoded in the absence of an immediate memory test. Consistent with this result, Craig and Dewar [31] have observed the wakeful rest effect on recognition memory performance for picture stimuli that were encoded via another semantic incidental orienting task (i.e., indoor vs. outdoor judgments) without including an immediate memory test. In light of the benefits observed in the response latency measures in Experiment 3, it is provocative to note that Craig and Dewar [31] also used pictorial stimuli, possibly reflecting a true consolidation effect in recognition memory. In any case, it appears that the presence of an immediate memory test and the intentional encoding instructions are not necessary to produce the wakeful rest effect in recall or recognition performance.

It is also noteworthy that the significant benefit of wakeful rest for recall was consistently observed across auditory and visual word stimuli, using different distractor tasks. In this regard, our results are inconsistent with suggestions that differences in the distractor task might moderate the presence of wakeful rest effects [33,34,35]. Specifically, prior authors have argued that a distractor task should only disrupt episodic enhancement for recent encoding if the task demands episodic processing, presumably driven by the hippocampal system. In contrast, tasks like the N-back working memory task arguably do not engage episodic processing, and hence, should not interfere with memory consolidation in comparison to a wakeful rest period. Indeed, this argument is supported by multiple prior experiments [33,34,35], but recent attempts to replicate this effect have demonstrated conflicting results in two experiments [36]. The interpretation that the wakeful rest effect should be modulated by the episodic demands of the distractor task is not supported by the current results. In Experiment 1, we used a simple 1-back digit-monitoring distractor task, similar to the N-back tasks used by Varma and colleagues [34,35], which should place minimal demand on hippocampal processing. In Experiment 2, we used a spot-the-difference distractor task, similar to one used by Dewar and colleagues [21], among others. Notably, other groups have argued that tasks like spot-the-difference might demand more complex processing, perhaps even involving autobiographical recall or future planning, as they feature concrete semantic cues, which may trigger autobiographical thinking [24,33,35,77]. Critically, in the present experiments, wakeful rest effects were observed for recall of verbal stimuli, regardless of the distractor task selected. In this sense, the present results are more consistent with King and Nicosia [36] than Varma and colleagues [34,35]. Nevertheless, future studies should compare different distractor types more systematically to clearly determine the boundary conditions of the wakeful rest effect.

### 5.3. Failure to Observe Wakeful Rest Effects in Recognition Memory

To our knowledge, only two previous experiments have reported significant wakeful rest effects in recognition memory performance. Specifically, Dewar and colleagues [25] found a wakeful rest effect in overall discriminability, as measured by *d’*, for both words and pronounceable nonwords. Additionally, as noted, Craig and Dewar [31] found that wakeful rest was also associated with increased discrimination of lures that were similar, but not identical, to incidentally encoded picture stimuli. In contrast to these effects, across all experiments in the current report (with the exception of the RT effect in Experiment 3), we found no evidence of a successful wakeful rest effect in any recognition task, regardless of the class of memory stimuli. In this sense, our results are more consistent with Richter and colleagues [39], who recently reported a null effect of wakeful rest on cued recognition assessments of contingency memory and evaluative conditioning.

The present task-specific benefits of wakeful rest might be expected by a theoretical account in which the wakeful rest effect is driven by strategic rehearsal, as opposed to consolidation. Although we attempted to limit strategic rehearsal via the incidental memory task, possibly we did not totally eliminate this contribution. Specifically, verbal rehearsal of the stimuli during the wakeful rest interval might particularly strengthen associations across words in the stimulus list. These intra-list associations might particularly aid performance in an unstructured free recall task, in which retrieval of one stimulus might serve as a retrieval cue for another stimulus from the same list. However, in a recognition task, which tests memory for stimuli in a random order, intermixing multiple source contexts, these associations might minimally influence performance (see, e.g., [78]).

Alternatively, it is possible that the benefits of wakeful rest might be limited to more self-generated or attentionally controlled retrieval processes, as demanded by recall tasks. Typically, recognition is less sensitive to these processes than recall tasks (see [79]), which indeed demonstrated significant wakeful rest effects in Experiments 1 and 2. Future studies might resolve this question by examining the impact of wakeful rest on process estimates of controlled and automatic retrieval processes. 

It is also possible that methodological differences across studies might contribute to the absence or presence of a wakeful rest effect in recognition performance. Specifically, both of the previous experiments that demonstrated significant wakeful rest effects in recognition used a between-participants design [25,31]. Interestingly, in each of these studies, the wakeful rest benefits on recognition performance were not driven by increased hits, but rather, by decreased false alarms to new foils [25] or increased discrimination of similar lures [31]. Unfortunately, our use of a within-participants manipulation of the intervening task does not allow us to compare false alarm rates between wakeful rest and distractor conditions, as each participant only responded to one set of foil trials on the final recognition task. Hence, our results suggest that wakeful rest does not produce an overall benefit in memory strength on a recognition task, but we cannot rule out the possibility that it might produce an increase in fine-grained discrimination of memory representations.

The failure to detect a wakeful rest effect across any of the recognition tests might also be driven by low sensitivity of the test. It is particularly surprising that robust wakeful rest effects were observed in recall, but not in a recognition task that immediately followed recall for those same words. One would have expected that those words that benefited in Experiment 1 and 2 from wakeful rest in recall would have produced superior recognition performance due to retrieval practice. It is possible that ceiling effects might obscure conditional differences in recognition performance. However, our use of multiple types of stimuli and different age groups resulted in a wide range of mean recognition performance values, especially see Experiment 3. Hence, the lack of a wakeful rest effect in the present recognition tests is not likely due to scaling issues.

## 6. Limitations

One general limitation of the present study is that we rely on the interpretation of null effects. This may be particularly difficult given the relatively small samples (particularly in Experiments 1 and 2), which limit power to detect main effect and interactive relationships. However, each of the null effects we consider are internally replicated in independent samples across multiple experiments, and moreover, are consistent when pooled across experiments, suggesting that the present null effects in the age by condition interaction are robust.

Further, our results imply that wakeful rest effects may depend on the format of the memory test, i.e., recall vs. recognition. However, as we did not anticipate this outcome, we did not design the study to properly test format in a factorial manner, i.e., recall always preceded recognition. As this design may introduce bias, e.g., completion of the recall test might affect performance on the following recognition test, future studies should confirm these results using a between-participants comparison of memory test format. However, as noted above, one would have expected a wakeful rest effect to benefit recognition in the current study, since the previous recall for the same words did produce a benefit of wakeful rest. Moreover, the recognition test in Experiment 3 for abstract shapes only produced an effect in speeded recognition performance only for the younger adults. Although intriguing, this pattern clearly needs further exploration. 

Finally, we tested age differences in the wakeful rest effect using a cross-sectional extreme groups design. Future studies might provide greater insight into how the wakeful rest effect changes in relation to aging processing by utilizing longitudinal designs and/or more continuous tests of age relationships [80], potentially even including children.

## 7. Conclusions

The present experiments contribute novel insights into the study of wakeful rest-dependent memory benefits. Wakeful rest has been viewed as an important behavioral paradigm to explore consolidation-like mechanisms, when the participant is not engaged in a specific task, a period which may produce mind-wandering. Because older adults produce less mind-wandering than younger adults and produce episodic memory deficits, we explored age differences in the wakeful rest effect. In three experiments, we have replicated and extended these effects in new directions, critically demonstrating that they are robustly observed in both younger and older adults for recall of incidentally encoded verbal stimuli. Additionally, our results suggest potentially important boundary conditions on the effect: specifically, that it appears to be robustly observed for recall, but not for recognition performance (using multiple measures). As discussed, this task-dependent finding is not predicted by a consolidation account and may suggest that the wakeful rest effect is partly driven by other processes, possibly including controlled rehearsal, which may have contributed to performance even in the present conservative design. In contrast, if indeed consolidation processes are being tapped by the wakeful rest period in recall performance, then the present results suggest that consolidation is preserved in older adults. Therefore, older adults may benefit more from interventions to support retrieval and encoding, rather than consolidation processes.

## Figures and Tables

**Table 1:** Demographic characteristics of the final samples in Experiments 1–3. Group difference *p* values are reported from independent-samples *t* tests for continuous variables or *χ*^2^ tests for categorical variables. Effect sizes of group differences are reported for continuous variables using *η^2^*. Experiment 1.

	Experiment 1				Experiment 2				Experiment 3			
	Younger, *n* = 24	Older, *n* = 16	*p* Value	*η* ^2^	Younger, *n* = 24	Older, *n* = 16	*p* Value	*η* ^2^	Younger, *n* = 32	Older, *n* = 32	*p* Value	*η* ^2^
**Age**			<0.001	0.93			<0.001	0.95			<0.001	0.94
*Mean (SD)*	20.9 (1.4)	64.2 (9.7)			20.7 (1.7)	66.0 (8.3)			20.0 (1.2)	63.7 (7.9)		
**Sex**			>0.9				0.7				>0.9	
*Female*	17 (71%)	12 (75%)			19 (79%)	11 (69%)			20 (62%)	20 (67%)		
*Male*	7 (29%)	4 (25%)			5 (21%)	5 (31%)			12 (38%)	10 (33%)		
**Education**			0.3	0.03			<0.001	0.36			<0.001	0.29
*Mean (SD)*	14.6 (1.4)	15.3 (2.6)			13.9 (1.3)	16.6 (2.4)			13.4 (1.2)	15.9 (2.7)		
**Race**			0.007				0.4				0.029	
*White*	13 (54%)	12 (75%)			15 (62%)	12 (75%)			20 (62%)	24 (80%)		
*Black or African American*	0 (0%)	3 (19%)			3 (12%)	2 (12%)			3 (9.4%)	5 (17%)		
*Asian*	10 (42%)	0 (0%)			4 (17%)	1 (6.2%)			8 (25%)	0 (0%)		
*More than one race*	1 (4.2%)	0 (0%)			0 (0%)	1 (6.2%)			0 (0%)	1 (3.3%)		
*Prefer not to respond*	0 (0%)	1 (6.2%)			2 (8.3%)	0 (0%)			1 (3.1%)	0 (0%)		

## Data Availability

Data supporting the reported results can be found at https://osf.io/jmx4b/.

## Supplementary Materials

The following supporting information can be downloaded at: https://www.mdpi.com/article/10.3390/brainsci12121609/s1, Figure S1: Barplots of corrected recall performance (proportion recalled - proportion intrusion), as a function of age group; Figure S2: Boxplots of proportion recalled ratio (wakeful rest / distractor condition), as a function of age group; Table S1: ANCOVA results from Experiment 2; Table S2: ANCOVA results from Experiment 3.

## References

[1] McGaugh J.L. (2000). Memory—A Century of Consolidation. Science.

[2] McGaugh J.L. (2015). Consolidating Memories. Annu. Rev. Psychol..

[3] Dudai Y. (2004). The Neurobiology of Consolidations, Or, How Stable Is the Engram?. Annu. Rev. Psychol..

[4] Frankland P.W., Bontempi B. (2005). The Organization of Recent and Remote Memories. Nat. Rev. Neurosci..

[5] Wixted J.T. (2004). The Psychology and Neuroscience of Forgetting. Annu. Rev. Psychol..

[6] Wixted J.T., Cai D.J. (2013). Memory Consolidation. The Oxford Handbook of Cognitive Neuroscience, Volume 1: Core Topics.

[7] Nadel L., Moscovitch M. (1997). Memory Consolidation, Retrograde Amnesia and the Hippocampal Complex. Curr. Opin. Neurobiol..

[8] Moscovitch M., Nadel L., Winocur G., Gilboa A., Rosenbaum R.S. (2006). The Cognitive Neuroscience of Remote Episodic, Semantic and Spatial Memory. Curr. Opin. Neurobiol..

[9] Yonelinas A.P., Ranganath C., Ekstrom A., Wiltgen B. (2019). A Contextual Binding Theory of Episodic Memory: Systems Consolidation Reconsidered. Nat. Rev. Neurosci..

[10] Squire L.R., Alvarez P. (1995). Reterograde Amnesia and Memory Consolidation: A Neurobiological Persepctive. Curr. Opin. Neurobiol..

[11] Squire L.R., Haist F., Shimamura A.P. (1989). The Neurology of Memory: Quantitative Assessment of Retrograde Amnesia in Two Groups of Amnesic Patients. J. Neurosci..

[12] Clark R.E., Broadbent N.J., Zola-Morgan S.M., Squire L.R. (2002). Anterograde Amnesia and Temporally Graded Retrograde Amnesia for a Nonspatial Memory Task after Lesions of Hippocampus and Subiculum. J. Neurosci..

[13] McClelland J.L., McNaughton B.L., O’Reilly R.C. (1995). Why There Are Complementary Learning Systems in the Hippocampus and Neocortex: Insights From the Successes and Failures of Connectionist Models of Learning and Memory. Psychol. Rev..

[14] Stickgold R. (2005). Sleep-Dependent Memory Consolidation. Nature.

[15] Gais S., Born J. (2004). Low Acetylcholine during Slow-Wave Sleep Is Critical for Declarative Memory Consolidation. Proc. Natl. Acad. Sci. USA.

[16] Rasch B.H., Born J., Gais S. (2006). Combined Blockade of Cholinergic Receptors Shifts the Brain from Stimulus Encoding to Memory Consolidation. J. Cogn. Neurosci..

[17] Ji D., Wilson M.A. (2007). Coordinated Memory Replay in the Visual Cortex and Hippocampus during Sleep. Nat. Neurosci..

[18] Tambini A., Ketz N., Davachi L. (2010). Enhanced Brain Correlations during Rest Are Related to Memory for Recent Experiences. Neuron.

[19] Wamsley E.J. (2019). Memory Consolidation during Waking Rest. Trends Cogn. Sci..

[20] Wamsley E.J. (2022). Offline Memory Consolidation during Waking Rest. Nat. Rev. Psychol..

[21] Dewar M.T., Alber J., Butler C., Cowan N., Della Sala S. (2012). Brief Wakeful Resting Boosts New Memories over the Long Term. Psychol. Sci..

[22] Alber J., Della Sala S., Dewar M.T. (2014). Minimizing Interference With Early Consolidation Boosts 7-Day Retention in Amnesic Patients. Neuropsychology.

[23] Brokaw K., Tishler W., Manceor S., Hamilton K., Gaulden A., Parr E., Wamsley E.J. (2016). Resting State EEG Correlates of Memory Consolidation. Neurobiol. Learn. Mem..

[24] Craig M., Della Sala S., Dewar M.T. (2014). Autobiographical Thinking Interferes with Episodic Memory Consolidation. PLoS ONE.

[25] Dewar M.T., Alber J., Cowan N., Della Sala S. (2014). Boosting Long-Term Memory via Wakeful Rest: Intentional Rehearsal Is Not Necessary, Consolidation Is Sufficient. PLoS ONE.

[26] Mercer T. (2015). Wakeful Rest Alleviates Interference-Based Forgetting. Memory.

[27] Wang S.Y., Baker K.C., Culbreth J.L., Tracy O., Arora M., Liu T., Morris S., Collins M.B., Wamsley E.J. (2021). “Sleep-Dependent” Memory Consolidation? Brief Periods of Post-Training Rest and Sleep Provide an Equivalent Benefit for Both Declarative and Procedural Memory. Learn. Mem..

[28] Craig M., Dewar M.T., Della Sala S., Wolbers T. (2015). Rest Boosts the Long-Term Retention of Spatial Associative and Temporal Order Information. Hippocampus.

[29] Craig M., Wolbers T., Harris M.A., Hauff P., Della Sala S., Dewar M.T. (2016). Comparable Rest-Related Promotion of Spatial Memory Consolidation in Younger and Older Adults. Neurobiol. Aging.

[30] Craig M., Dewar M.T., Harris M.A., Della Sala S., Wolbers T. (2015). Wakeful Rest Promotes the Integration of Spatial Memories into Accurate Cognitive Maps. Hippocampus.

[31] Craig M., Dewar M.T. (2018). Rest-Related Consolidation Protects the Fine Detail of New Memories. Sci. Rep..

[32] Humiston G.B., Wamsley E.J. (2018). A Brief Period of Eyes-Closed Rest Enhances Motor Skill Consolidation. Neurobiol. Learn. Mem..

[33] Martini M., Sachse P. (2020). Factors Modulating the Effects of Waking Rest on Memory. Cogn. Process.

[34] Varma S., Takashima A., Krewinkel S., van Kooten M., Fu L., Medendorp W.P., Kessels R.P.C., Daselaar S.M. (2017). Non-Interfering Effects of Active Post-Encoding Tasks on Episodic Memory Consolidation in Humans. Front. Behav. Neurosci..

[35] Varma S., Daselaar S.M., Kessels R.P.C., Takashima A. (2018). Promotion and Suppression of Autobiographical Thinking Differentially Affect Episodic Memory Consolidation. PLoS ONE.

[36] King O., Nicosia J. (2022). The Effects of Wakeful Rest on Memory Consolidation in an Online Memory Study. Front. Psychol..

[37] Martini M., Riedlsperger B., Maran T., Sachse P. (2017). The Effect of Post-Learning Wakeful Rest on the Retention of Second Language Learning Material over the Long Term. Curr. Psychol..

[38] Humiston G.B., Tucker M.A., Summer T., Wamsley E.J. (2019). Resting States and Memory Consolidation: A Preregistered Replication and Meta-Analysis. Sci. Rep..

[39] Richter J., Seffen A., Benedict T., Gast A. (2021). No Evidence of Consolidation of Evaluative Conditioning during Waking Rest and Sleep. Cogn. Emot..

[40] Craik F.I.M., Byrd M., Craik F.I.M., Trehub S. (1982). Aging and Cognitive Deficits. Aging and Cognitive Processes.

[41] Craik F.I.M., Jennings J.M., Craik F.I.M., Salthouse T.A. (1992). Human Memory. The Handbook of Aging and Cognition.

[42] Balota D.A., Dolan P.O., Duchek J.M., Tulving E., Craik F.I.M. (2000). Memory Changes in Healthy Young and Older Adults. The Oxford Handbook of Memory.

[43] Martini M., Martini C., Bernegger C., Sachse P. (2018). Post-Encoding Wakeful Resting Supports the Retention of New Verbal Memories in Children Aged 13–14 Years. Br. J. Dev. Psychol..

[44] Martini M., Zamarian L., Sachse P., Martini C., Delazer M. (2018). Wakeful Resting and Memory Retention: A Study with Healthy Older and Younger Adults. Cogn. Process.

[45] Sacripante R., McIntosh R.D., Della Sala S. (2019). Benefit of Wakeful Resting on Gist and Peripheral Memory Retrieval in Healthy Younger and Older Adults. Neurosci. Lett..

[46] Varma S., Takashima A., Fu L., Kessels R.P.C. (2019). Mindwandering Propensity Modulates Episodic Memory Consolidation. Aging Clin. Exp. Res..

[47] Nicosia J., Balota D.A. Targeted Memory Reactivation and Consolidation-like Processes during Mind-Wandering in Younger and Older Adults. J. Exp. Psychol. Learn. Mem. Cogn..

[48] Giambra L.M. (1989). Task-Unrelated-Thought Frequency as a Function of Age: A Laboratory Study. Psychol. Aging.

[49] Jackson J.D., Balota D.A. (2012). Mind-Wandering in Younger and Older Adults: Converging Evidence from the Sustained Attention to Response Task and Reading for Comprehension. Psychol. Aging.

[50] Jordaõ M., Ferreira-Santos F., Pinho M.S., St Jacques P.L. (2019). Meta-Analysis of Aging Effects in Mind Wandering: Methodological and Sociodemographic Factors. Psychol. Aging.

[51] Martini M., Martini C., Maran T., Sachse P. (2018). Effects of Post-Encoding Wakeful Rest and Study Time on Long-Term Memory Performance. J. Cogn. Psychol..

[52] Martini M., Marhenke R., Martini C., Rossi S., Sachse P. (2020). Individual Differences in Working Memory Capacity Moderate Effects of Post - Learning Activity on Memory Consolidation over the Long Term. Sci. Rep..

[53] McCabe D.P., Roediger H.L., McDaniel M.A., Balota D.A., Hambrick D.Z. (2010). The Relationship between Working Memory Capacity and Executive Functioning: Evidence for a Common Executive Attention Construct. Neuropsychology.

[54] Roediger H.L., Karpicke J.D. (2006). Test-Enhanced Learning: Taking Memory Tests Imporves Long-Term Retention. Psychol. Sci..

[55] Balota D.A., Neely J.H. (1980). Test-Expectancy and Word-Frequency Effects in Recall and Recognition. J. Exp. Psychol. Hum. Learn. Mem..

[56] Hartley T., Houghton G. (1996). A Linguistically Constrained Model of Short-Term Memory for Nonwords. J. Mem. Lang..

[57] Multhaup K.S., Balota D.A., Cowan N. (1996). Implications of Aging, Lexicality, and Item Length for Mechanisms Underlying Memory Span. Psychon. Bull. Rev..

[58] Katzman R., Brown T., Fuld P., Peck A., Schechter R., Schimmel H. (1983). Validation of a Short Orientation-Memory-Concentration Test of Cognitive Impairment. Am. J. Psychiatry.

[59] Morris J.C., Heyman A., Mohs R.C., Hughes J.P., van Belle G., Fillenbaum G., Mellits E.D., Clark C. (1989). The Consortium to Establish a Registry for Alzheimer’s Disease (CERAD). Part I. Clinical and Neuropsychological Assessment of Alzheimer’s Disease. Neurology.

[60] Balota D.A., Yap M.J., Cortese M.J., Hutchison K.A., Kessler B., Loftis B., Neely J.H., Nelson D.L., Simpson G.B., Treiman R. (2007). The English Lexicon Project. Behav. Res. Methods.

[61] Salthouse T.A., Birren J.E., Schaie K.W. (1985). Speed of Behavior and Its Implications for Cognition. Handbook of the Psychology of Aging.

[62] Cerella J., Birren J.S., Schaie K.W. (1990). Aging and Information-Processing Rate. Handbook of the Psychology of Aging.

[63] Faust M.E., Balota D.A., Spieler D.H., Ferraro F.R. (1999). Individual Differences in Information-Processing Rate and Amount: Implications for Group Differences in Response Latency. Psychol. Bull..

[64] R Core Team (2021). R: A Language and Environment for Statistical Computing.

[65] Penney C.G. (1989). Modality Effects and the Structure of Short-Term Verbal Memory. Mem. Cognit..

[66] Ginns P. (2005). Meta-Analysis of the Modality Effect. Learn. Instr..

[67] Lindner K., Blosser G., Cunigan K. (2009). Visual versus Auditory Learning and Memory Recall Performance on Short-Term versus Long-Term Tests. Mod. Psychol. Stud..

[68] Vanderplas J.M., Garvin E.A. (1951). The Association Value of Random Shapes. J. Exp. Psychol..

[69] Jackson J.J., Bogg T., Walton K.E., Wood D., Harms P.D., Lodi-Smith J., Edmonds G.W., Roberts B.W. (2009). Not All Conscientiousness Scales Change Alike: A Multimethod, Multisample Study of Age Differences in the Facets of Conscientiousness. J. Pers. Soc. Psychol..

[70] Nicosia J., Balota D.A. (2021). Dispositional Factors Account for Age Differences in Self-Reported Mind-Wandering. Psychol. Aging.

[71] Perfect T.J., Dasgupta Z.R.R. (1997). What Underlies the Deficit in Reported Recollective Experience in Old Age?. Mem. Cogn..

[72] Hertzog C., McGuire C.L., Lineweaver T.T. (1998). Aging, Attributions, Perceived Control, and Strategy Use in a Free Recall Task. Aging, Neuropsychol. Cogn..

[73] Naveh-Benjamin M., Brav T.K., Levy O. (2007). The Associative Memory Deficit of Older Adults: The Role of Strategy Utilization. Psychol. Aging.

[74] Kirchhoff B.A., Anderson B.A., Barch D.M., Jacoby L.L. (2012). Cognitive and Neural Effects of Semantic Encoding Strategy Training in Older Adults. Cereb. Cortex.

[75] Faul F., Erdfelder E., Lang A.-G., Buchner A. (2007). G*Power 3: A Flexible Statistical Power Analysis Program for the Social, Behavioral, and Biomedical Sciences. Behav. Res. Methods.

[76] Hasher L., Zacks R.T., Bower G. (1988). Working Memory, Comprehension, and Aging: A Review and a New View. The Psychology of Learning and Motivation.

[77] Berntsen D. (1996). Involuntary Autobiographical Memories. Appl. Cogn. Psychol..

[78] Neely J.H., Balota D.A. (1981). Test-Expectancy and Semantic-Organization Effects in Recall and Recognition. Mem. Cognit..

[79] Craik F.I.M., Klix F., Hagendorf H. (1986). A Functional Account of Age Differences in Memory. Human Memory and Cognitive Capabilities: Mechanisms and Performances.

[80] Salthouse T.A. (2000). Methodological Assumptions in Cognitive Aging Research. Handbook of Aging and Cognition.

