# Supplementary material for: Wakeful Rest Benefits Recall, but Not Recognition, of Incidentally Encoded Memory Stimuli in Younger and Older Adults
Source: Brain Sci. 2022 Nov 24;12(12):1609. doi: 10.3390/brainsci12121609 (PMC9775546; doi:10.3390/brainsci12121609)
Supplement: Supplementary file 1 [file brainsci-12-01609-s001.zip › brainsci-1956771-supplementary.pdf]

Supplementary Materials
Intrusion-Corrected Recall Performance

The primary recall analyses in Experiments 1 and 2 do not account for differing intrusion rates of incorrect, unstudied words during the recall task. In these supplementary analyses, we analyzed corrected recall performance by subtracting the proportion of intrusion items from the proportion of correctly recalled items. We then applied the same ANOVA test to these corrected recall performance measures.

In Experiment 1, this analysis revealed a significant main effect of age on corrected recall, $F(1,38) = 8.84$, $p = .005$, $\eta_G^2 = .14$ and a significant main effect of condition, $F(1,38) = 6.64$, $p = .014$, $\eta_G^2 = .05$. There was no evidence of an interaction between age and condition, $F(1,38) = 0.05$, $p = .816$, $\eta_G^2 < .001$. As shown in Figure S1A, older adults had lower corrected recall rates than younger adults, and both groups had greater corrected recall rates in the wakeful rest condition than in the spot the difference condition. All of these results were consistent with the primary results using the proportion of recalled items.

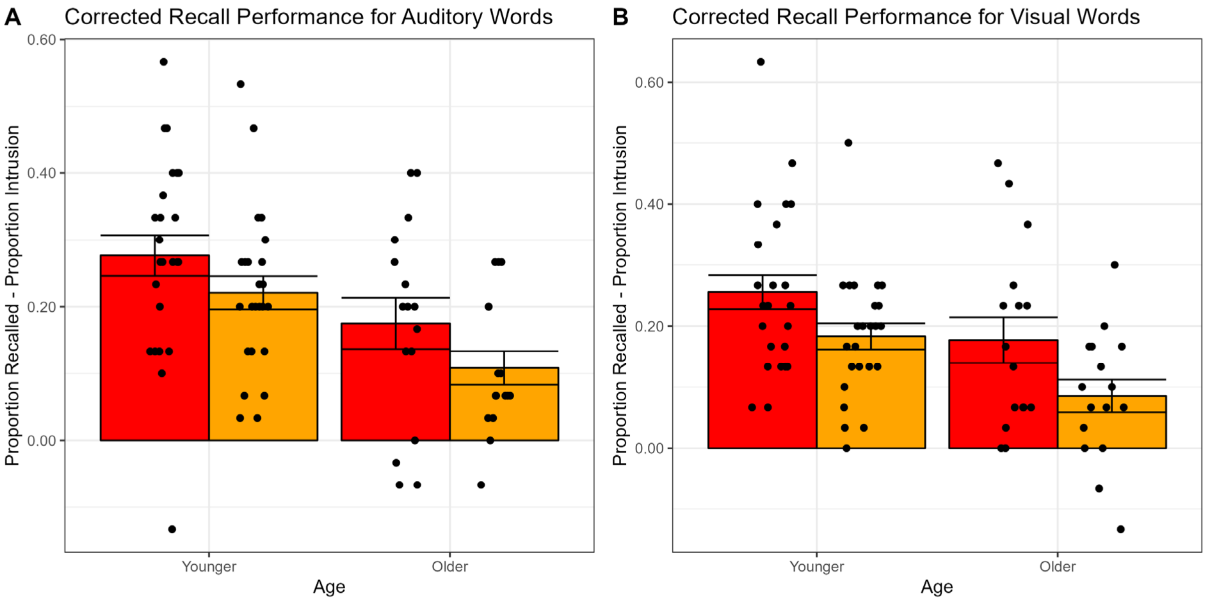

Figure S1. Barplots of corrected recall performance (proportion recalled - proportion intrusion), as a function of age group. (**A**) Auditory word stimuli in Experiment 1. (**B**) Visual word stimuli in Experiment 2. Bar heights reflect mean values. Points reflect individual participants. Error bars are standard error of the mean.

In Experiment 2, this analysis revealed a significant main effect of age on corrected recall, $F(1,38) = 6.34$, $p = .016$, $\eta_G^2 = .11$ and a significant main effect of condition, $F(1,38) = 16.89$, $p < .001$, $\eta_G^2 = .10$. There was no evidence of an interaction between age and condition, $F(1,38) = 0.24$, $p = .627$, $\eta_G^2 = .002$. As shown in Figure S1B, older adults had lower corrected recall rates than younger adults, and both groups had greater corrected recall rates in the wakeful rest condition than in the spot the difference condition. All of these results were consistent with the primary results using the proportion of recalled items.

Relative Recall Performance

It is possible that the wakeful rest period might facilitate recall of a certain percentage of previously studied items. As the two age groups differed in recall performance in the distractor condition, age differences in the wakeful rest effect might be more appropriately expressed as the ratio of correct recall in the wakeful rest condition, as compared to the distractor condition. This measure quantifies the degree to which recall performance is facilitated by the wakeful rest condition, relative to the distractor. Scores near 1 imply no facilitation, with increasing scores implying greater facilitation. Age differences in this relative recall performance ratio were tested using a one-way ANOVA, with age group as the only between-participants factor (younger vs. older adults).

In Experiment 1, this analysis revealed no significant main effect of age on the recall ratio measure, $F(1,38) = 0.08$, $p = .781$, $\eta_G^2 = .002$. As shown in Figure S2A, younger and older adults did not differ in the proportion to which the wakeful rest condition facilitated recall, relative to the distractor task.

In Experiment 2, this analysis revealed no significant main effect of age on the recall ratio measure, $F(1,36) = 0.28$, $p = .602$, $\eta_G^2 = .008$. As shown in Figure S2B, younger and older adults did not differ in the proportion to which the wakeful rest condition facilitated recall, relative to the distractor task.

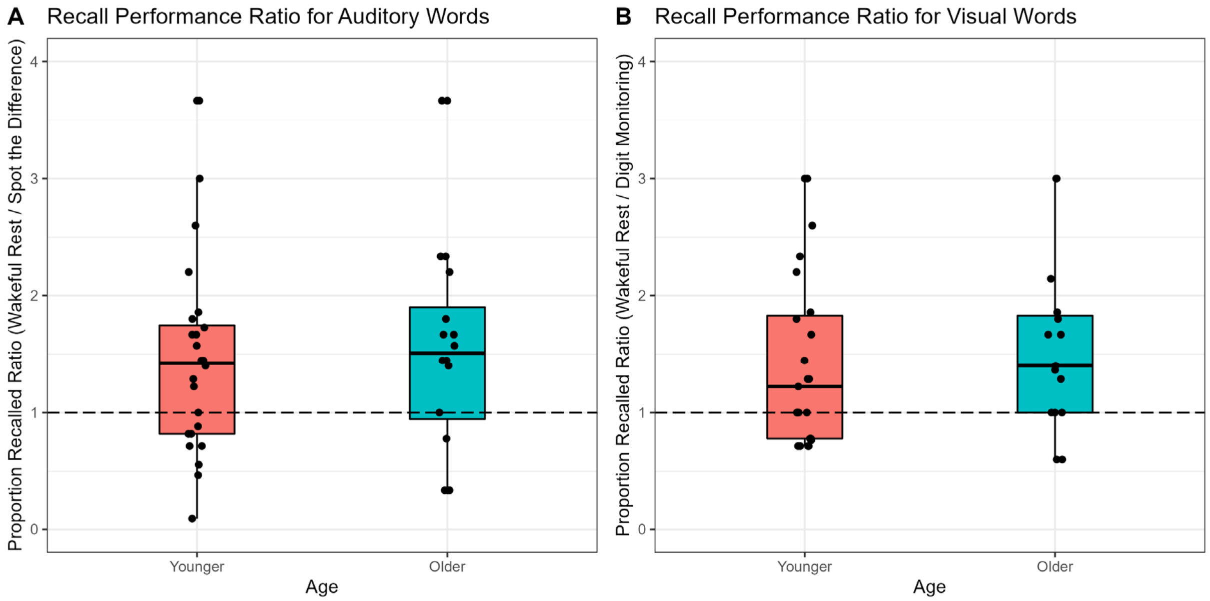

Figure S2. Boxplots of proportion recalled ratio (wakeful rest / distractor condition), as a function of age group. (**A**) Auditory word stimuli in Experiment 1. (**B**) Visual word stimuli in

Experiment 2. Points reflect individual participants. Dashed line reflects equal performance between wakeful rest and distractor conditions.

Controlling for Education

Since age groups in Experiments 2 and 3 differed in years of education, we also tested the effects of interest using supplementary ANCOVAs, which included education as a covariate of non-interest. Full results from these ANCOVAs are provided in the tables below.

Table S1. ANCOVA results from Experiment 2. All models include years of education as a continuous covariate of non-interest. DV = dependent variable.

DV	*Effect*	*F*	*p*	η_G^2
Recall	Age	2.72	0.108	0.050
**Recall**	**Condition**	**17.03**	**0.000**	**0.116**
Recall	Age:Condition	0.16	0.689	0.001

DV	*Effect*	*F*	*p*	η_G^2
Hits - FA	Age	0.87	0.358	0.020
Hits - FA	Condition	0.01	0.922	0.000
Hits - FA	Age:Condition	0.01	0.906	0.000

DV	*Effect*	*F*	*p*	η_G^2
Confidence	Age	0.24	0.624	0.006
Confidence	Condition	0.00	0.952	0.000
Confidence	Age:Condition	0.02	0.898	0.000

DV	*Effect*	*F*	*p*	η_G^2
ZRT	Age	0.34	0.561	0.000
ZRT	Condition	1.24	0.272	0.032
ZRT	Age:Condition	0.11	0.744	0.003

Table S2. ANCOVA results from Experiment 3. All models include years of education as a continuous covariate of non-interest. DV = dependent variable.

DV	*Effect*	*F*	*p*	η_G^2
**Hits - FA**	**Age**	**11.29**	**0.001**	**0.124**
Hits - FA	Condition	0.05	0.832	0.000
Hits - FA	Age:Condition	0.75	0.389	0.004

DV	*Effect*	*F*	*p*	η_G^2
Confidence	Age	2.42	0.125	0.033
Confidence	Condition	0.17	0.680	0.001
Confidence	Age:Condition	4.00	0.050	0.014

DV	*Effect*	*F*	*p*	η_G^2
ZRT	Age	0.01	0.922	0.000
**ZRT**	**Condition**	**4.56**	**0.037**	**0.072**
**ZRT**	**Age:Condition**	**4.74**	**0.034**	**0.075**
